# Supplementary material for: A yeast phenomic model for the influence of Warburg metabolism on genetic buffering of doxorubicin
Source: Cancer Metab. 2019 Oct 23;7:9. doi: 10.1186/s40170-019-0201-3 (PMC6806529; doi:10.1186/s40170-019-0201-3)
Supplement: Supplementary file 9 — Additional file 9. GO term-specific heatmaps for REMc/GTF-enriched clusters. GO term-specific heatmaps for significant GO process terms were generated as described in methods and Figs. 3 and 4. Any related child terms are presented in subsequent pages of the parent file name. GO terms with more than 100 children, with 2 or fewer genes annotated to the term, or a file size over 300KB are not shown. All heatmaps are generated with the same layout (see Figs. 3 and 4). [file 40170_2019_201_MOESM9_ESM.bz2 › Additional_File9_GOTermHeatmaps/Additional_File9_GOTermHeatmap/positive_regulation_of_transcription_elongation_from_RNA_polymerase_II_promoter.pdf]

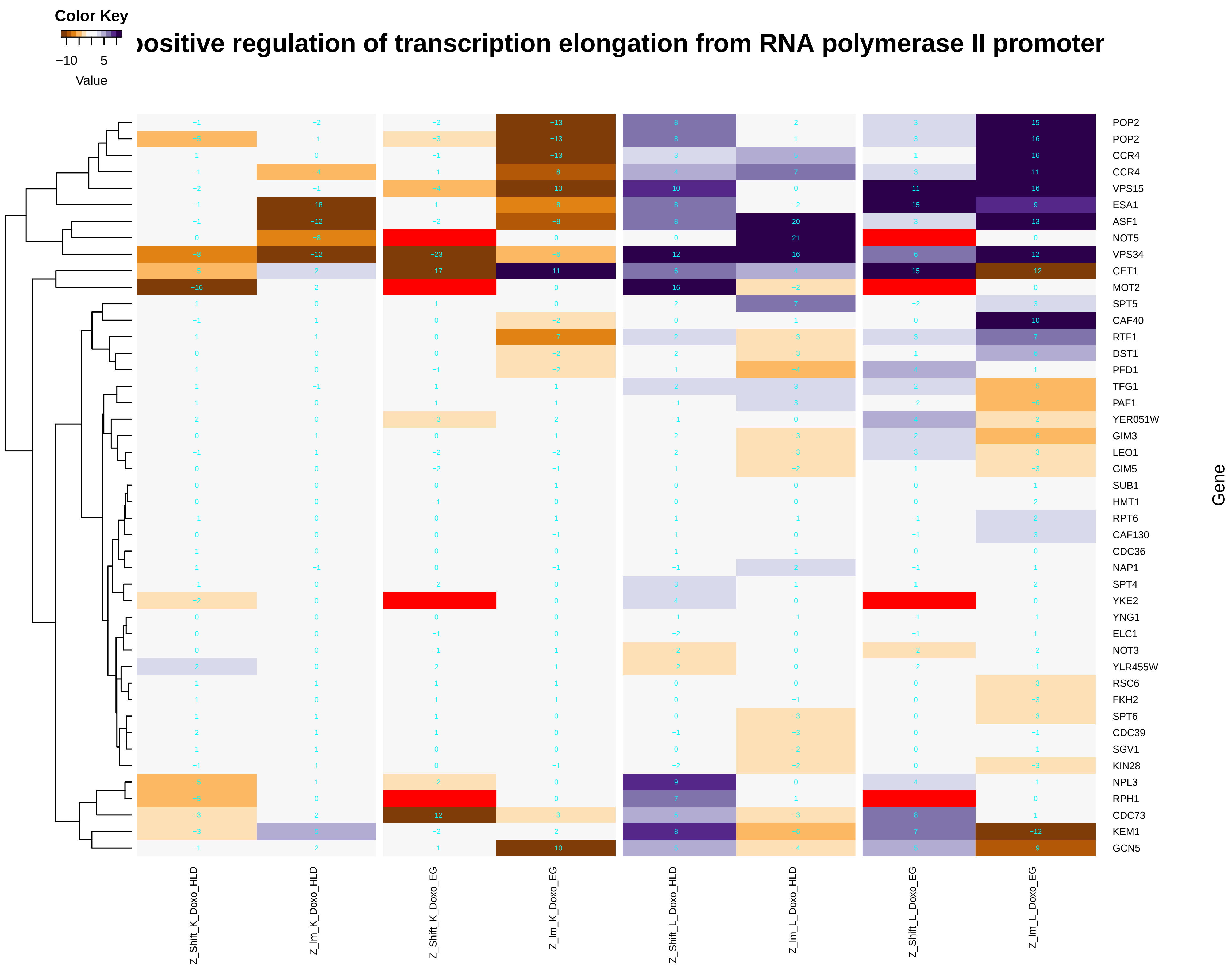

Color Key
−10
5
Value
positive regulation of transcription elongation from RNA polymerase II promoter
POP2
POP2
CCR4
CCR4
VPS15
ESA1
ASF1
NOT5
VPS34
CET1
MOT2
SPT5
CAF40
RTF1
DST1
PFD1
TFG1
PAF1
YER051W
GIM3
LEO1
GIM5
SUB1
HMT1
RPT6
CAF130
CDC36
NAP1
SPT4
YKE2
YNG1
ELC1
NOT3
YLR455W
RSC6
FKH2
SPT6
CDC39
SGV1
KIN28
NPL3
RPH1
CDC73
KEM1
GCN5
Gene
Z_Shift_K_Doxo_HLD
Z_Im_K_Doxo_HLD
Z_Shift_K_Doxo_EG
Z_Im_K_Doxo_EG
Z_Shift_L_Doxo_HLD
Z_Im_L_Doxo_HLD
Z_Shift_L_Doxo_EG
Z_Im_L_Doxo_EG
